# Medicine

**Published:** 1897-12

**Authors:** R. Shingleton Smith


### Progress of the Medical Sciences.

### MEDICINE.

The two great autumnal medical meetings of this year, the British and the International, may naturally be expected to furnish much material for a report on the progress of medicine. The general tenor of the Montreal Meeting of the British Medical Association is well indicated in the following quotation from Dr. William Osler's address on the factors which have moulded the profession in English-speaking lands beyond the narrow seas—of British medicine in Greater Britain: "Meanwhile to the throbbing vitality of modern medicine the two great meetings held this month, in lands so widely distant, bear eloquent testimony. Free, cosmopolitan, no longer hampered by the dogmas of schools, we may feel a just pride in a profession almost totally emancipated from the bondage of error and prejudice. . . . There remains for us, Greater Britons, of whatsoever land, the bounden duty to cherish the best traditions of our fathers, and particularly of the men who gave to British medicine its most distinctive features; of the men, too, who found for us the light and liberty of Greek thought—Linacre, Harvey, and Sydenham—those 'ancient founts of inspiration,' and models for all time in literature, science and practice."

Several of the other general addresses were on similar topics relating to the evolution and history of different branches of the profession. "The Work of Pasteur and the Modern Conception of Medicine," by Prof. Charles Richet, was a very fitting address as part of a meeting which was distinguished by (perhaps more than anything else) a more or less continuous ovation of Lord Lister, whose work has been a direct outcome of the teachings of Pasteur. The address on public medicine by Dr. Hermann M. Biggs gave us the most advanced sequel to the work of Pasteur and of Lister. It is certainly remarkable that a democratic country should have adopted the most autocratic and paternal methods for the suppression of everything detrimental to the public health, and that it appears to be possible to adopt measures more arbitrary in many respects than could be adopted in most other countries, simply because the government is democratic. Dr. Stephen Mackenzie's interesting summary of the influences that have determined the progress of medicine during the preceding two and a half centuries pointed out the fact that human life has been prolonged and several years added to the most useful and valuable period of life, that there has been a manifest decrease of mortality from small-pox, scarlet fever, diarrhœa, typhus, enteric fever, phthisis, convulsions, croup, diseases of the digestive system, and puerperal diseases; and that, although there has been an *increase* of mortality from cancer and some other diseases, yet the gains have been greater than the losses.

In the medical section a debate on the relation of **rheumatoid arthritis** to diseases of the nervous system, tuberculosis, and rheumatism, was opened by Dr. James Stewart, of Montreal, who from an analysis of forty cases, extending over three and a half years, concluded as follows: Rheumatoid arthritis is a disease prone to occur in people of a rheumatic tendency and who have suffered from subacute rheumatic attacks. The presence of infectious disease of any kind tends to increase this tendency, as does the operation of all causes having a depressing influence on the resisting power of the nervous system (worry, exposure to cold, traumatism). There is no sharp dividing line between certain cases of chronic rheumatism and the earlier stages of rheumatoid arthritis. There is not sufficient evidence to support the views commonly held as to the nervous origin of rheumatoid arthritis. There is no direct relationship between tuberculosis and rheumatoid arthritis. The polyarticular forms of rheumatoid arthritis have clinically the features of an infectious disease; and the results of recent investigations point very strongly to its infectious nature.

In the discussion which followed I directed attention to a recent debate on this subject, opened by Dr. Bäumler (Freiburg) at the German Congress (Berlin, 1897), when the name chronic articular or polyarticular rheumatism appeared to be most in favour. I ventured to claim that the disease was not a rheumatism at all, but should be designated as a polyarticular arthritis, and that it was likely to be due to some infective agent, that it bore no relation to either tubercle or syphilis, but that its close pathological association with post-gonorrhœal arthritis, in which the disease had been clearly proved to be due to the gonococcus, rendered it likely that some such parasitic organism as that described by Bannatyne, Wohlmann, and Blaxall,[1] was the infective agent of the disease, and that all other of the numerous etiological factors commonly described might be swept away. I pointed out the differences between the early and late stages of the disease, and thought it probable that the parasitic stage was of limited duration, and that the later phenomena were all the sequelæ of the arthritis, in which perhaps mixed and secondary infections might play a part. I alluded to a case under my observation, occurring in a girl aged 16, of previous good health and of good family history, who was now the victim of numerous deformities from a very acute polyarticular arthritis of two years ago, but from which we may exclude all questions relating to diet, alcohol, emotion, shock, damp and cold, injury, even lowered vitality; but where a general rheumatic or pyæmic toxæmia seemed to be a far more probable explanation. The dissociation of the primary attack from the sequelæ of the disease seemed essential with regard to treatment: little attempt had yet been made to modify the former, whereas it would seem obvious that this is the time when every effort should be made to arrest the mischief before any serious structural defect had developed: the salicylates have proved themselves almost inert in this disease; but it remains to be proved whether some such drugs as the carbonate of guaiacol in full doses may be of more service.

Dr. Stewart stated that the most successful method of treatment was by baths of various kinds; but one fails to see what good such treatment can do, or how it could be borne in the acute stages of arthritis: this, like most of the other classical methods of treatment, appears to be more suited for the chronic stages of the malady. In the further development of the discussion, the relationship to nerve disease was emphasised by Dr. Lindsay (Belfast), Dr. Osler (Baltimore), and Dr. Jacobi (New York); but the general conclusion left on the minds of the audience must have been in accord with Dr. Stewart's dictum

[1] *Lancet*, 1896, i. 1120.

that the theory of the nervous origin of rheumatoid arthritis is not supported by sufficient evidence.

At the Moscow meeting[1] one of the most interesting topics discussed was **the high altitude treatment of tuberculosis,** introduced by Dr von Ziemssen, who considered that of all the motley conglomeration of methods of treatment, all may be discarded with the exception of two ; viz., tuberculin and climate. Koch's old tuberculin has been forgotten; the value of the new has yet to be proved, and most people already doubt its efficacy. For the pulmonary form nothing has really been done; although other forms, such as lupus, have without doubt been cured. Some of those forms have their specifics; for tuberculin will often cure lupus, and iodoform will cure tuberculosis of joints: but we have as yet no specific for tuberculosis of the lungs.

There remains, therefore, only the climatic treatment. Of late the value of this has been clearly demonstrated. The best results are obtained when a patient is sent to a place near his own home; for a long journey is always depressing, and the comfort of seeing friends, especially to a sick man, is very great. The stage of the disease should determine this question; for if the case is much advanced, the patient should be kept as near home as possible.

Dr. Senator had tried tuberculin in a dozen cases, but without good results. The only treatment of value was the climatic. He questioned if the good was done by increasing the number of red corpuscles, as he had seen more than one case in which elevation had no visible influence on the corpuscular element, and yet the patient improved. Pharyngeal and laryngeal cases are to be regarded as a positive contra-indication to high altitude treatment of tuberculosis. Dr. von Leyden stated that in his hands the specific treatment had failed. The only treatment which had given positive results was the climatic. A patient should not be sent far from home; for the nearer he is to friends the more comfortable and contented he will be. Dr. Körnig had found an increase in the red blood-cells in patients who had gone to high altitudes, and he attributed to this increase a large part of the benefit derived.

The polycythæmia of those living at high altitudes is well known to increase with the elevation. The following table, given by Köppe,[2] is well worthy of perusal:

| Place. | Height. | Red Cells. | Author. |
|---|---|---|---|
| Christiania | 0 | 4,974,000 | Laache. |
| Göttingen | 148 metres | 5,225,000 | Schafter. |
| Tübingen | 314 " | 5,322,000 | Reinert. |
| Zürich | 414 " | 5,752,000 | Stierlin. |
| Auerbach | 425 " | 5,748,000 | Köppe. |
| Reiboldsgrün | 700 " | 5,900,000 | " |
| Arosa | 1,800 " | 7,000,000 | Egger. |
| The Cordilleras | 4,392 " | 8,000,000 | Viault. |

[1] *Med. Rec.*, 1897, lii. 394. [2] *Munchener med. Wchnschr.*, 1890, No. 41.

This fact gives us at once an argument in favour of the utility of high altitudes in the treatment of anæmia, and in all other conditions, more especially phthisis, in which anæmia is usually more or less pronounced; and it appears to be probable that the comparative immunity from phthisical contagion in those regions is due to the excellent sanguification of the patients who reside there.

Prof. E. von Leyden, of Berlin, in his paper at Moscow on the present **modes of treating consumptives, and their State control**, comments on the great importance of nourishment in liberal supply. Experience has shown that the more food the consumptive takes and digests the better are his chances of recovery.[1] He also, "while valuing highly the climatic advantages of high altitudes . . . warned his hearers that there is no actual immunity against tuberculosis in these regions, and we cannot depend upon any climate as in itself directly curative of this disease." He looked upon the assumed specific action of fresh air in tuberculosis as one of the myths of medicine. It has no such action; but its value is a purely hygienic one, since it aids greatly in strengthening the organism and rendering it more resistant to the attacks of pathogenic organisms. "The air should be pure, as free as possible from dust, not liable to great and sudden changes of temperature, and the place should be free from violent storms."

The fresh air and overfeeding plan of treatment, as practised at Nordach, Görbersdorf, and other sanatoria, has received much attention of late; and Dr. Volland, of Davos-Dorf, has drawn attention to the harm which may arise from a blind adherence to such methods. He believes that the disease is not to be driven back by forced feeding, but that dilatation and gastroptosis are likely to arise from systematic overfilling of the stomach. As regards the air cure, Dr. Volland believes in the old-fashioned idea that houses have been built, not only as shelters for the healthy against heat, cold, the vicissitudes of the weather in general, and the bad influence of night air, but, above all, for the sick, to whom such shelter is particularly necessary. Dr. Hector Mackenzie,[2] whilst agreeing in the main with Dr. Volland's protest, considers that much can be done for phthisical patients by a judicious dietary, and by encouraging them, as far as practicable, to lead an open-air life.

This is done as far as possible in the Adirondack Cottage Sanitarium at Saranac Lake, New York, which I had an opportunity of visiting recently. This charming little village, situated on one of the slopes of the Adirondacks, consists of a number of detached cottages, each of which accommodates only two or four patients, who live with doors and windows freely open, under excellent climatic conditions and surrounded by the most lovely scenery. From the report of the president, Dr. Edward L. Trudeau, for the year 1896, we learn that the results for

[1] *Med. Rec.*, 1897, lii. 354. [2] *Practitioner*, 1897, lix. 406.

the year were briefly as follows: Number of patients, 106. Discharged apparently cured, 24; with disease arrested, 37; improved, 21; unimproved or failed, 24. In this sanitarium, treatment by injections of modified tuberculin, in conjunction with the usual open-air and climatic plan, has been carried on extensively, with the following results: Of 14 patients so treated during the year there were: apparently cured, 6; the disease arrested, 6; improved, 1; failed, 1. And of the 24 patients treated in this way who have been discharged as "apparently cured" in the past five years, 22 are known to have remained well up to date, although many of them have returned to their former occupations and surroundings. Two have died during the past year: one in an insane asylum from some cerebral disease and without any pulmonary symptoms, the other of chronic alcoholism.

Dr. Vivant,[1] of Monte Carlo, after pointing out that no climate gives absolute immunity, remarked that the stations on the Riviera, the plains of the deserts, the high altitude resorts, all offer in different degrees all the active elements in climatic treatment; but the plains and the deserts offer no shelter from the heavy winds, the principal agents of colds and principal disseminators of dust. The Mediterranean resorts offer the advantage over the mountains that, with a superior solar radiation, they are less cold, permitting the patient to live in the open air continually in light thin clothes, giving the air and light the best chance to exert their favourable therapeutic action.

Dr. Whittaker, in a paper on "Generalisations from Six Years use of Tuberculin,"[2] maintains that the highest value of tuberculin is its diagnostic value, which is supreme, and which enables us to distinguish the disease at the start as a tuberculosis, before the development of sepsis or other complications which go to make up that composite picture which we call phthisis. Dr. Trudeau, of Saranac Lake, New York,[3] comments on the utility of the tuberculin test in incipient and suspected pulmonary tuberculosis. He states that "the differentiation of an inflammatory bronchitis of chronic type from pulmonary phthisis, of tuberculous pleuritis from the other forms of the affection, of an unresolved pneumonic process from a cheesy pneumonia, of simple inflammatory pyelitis and cystitis from the tuberculous variety of these diseases, and of a gonorrheal or rheumatic from a tuberculous synovitis, is often very difficult, and much light as to the exact nature of the process under observation may be expected by a careful application of the tuberculin test to such cases." In seven cases out of fourteen there was more or less reaction. Of the seven which did not react, two passed from observation, but the others remained well. The initial dose should be .5 mg., and he had never used a higher

1 *Med. Rec.*, 1897, lii. 395. 2 *Brit. M. J.*, 1897, ii. 1053.

3 *Med. News*, 1897, lxx. 687.

dose than 3 mg. Continental authorities generally agree as to the high diagnostic value of the tuberculin test.

The subject of "acquired immunity" is much discussed. Two kinds of immunity are now recognised—one against toxins produced by bacilli, one against the bacilli themselves; immunity, to be complete, should combine both these elements. Dr. G. Archdall Reid[1] remarks that "against some zymotic diseases—*e.g.*, small-pox and measles—an average individual is able, through illness and recovery, to acquire life-long immunity; against other diseases—*e.g.*, diphtheria and relapsing fever—immunity may be acquired, but is of limited duration only, and in a shorter or longer time the individual, lapsing his acquired powers of making resistance, becomes as liable to infection as ever he was; against a third class of diseases—*e.g.*, tuberculosis and leprosy—immunity can never be acquired."

Koch in his original paper raises the question whether, as regards tuberculosis, a natural immunity is possible. Apparently not; for "the malady may go on for years without any sign of immunity appearing; and even when arrested it is liable to break out afresh." Other facts tend to show that immunity is possible, but is only possible after absorption or digestion of the bacilli: no immunity commonly occurs because the bacilli are not absorbed at all. In acute tuberculosis, and experimental tuberculosis in guinea-pigs, there is a stage in which the bacilli, at one time in abundance, become so few that they are difficult to find—is this due to a process of immunisation too late to be of benefit? Attempts to produce this condition of immunity by artificial inoculation with either living or dead cultures have altogether failed. How to bring about, artificially, that inundation of the body with the poison contained in bacilli as occurs in acute tuberculosis, and so produce an immunity to them without harm to the body itself, is the problem which Koch has tried to solve.

The new tuberculin,[2] TR, contains all the immunising factors of the cultures of the bacilli, and guinea-pigs are readily rendered immune to virulent cultures, whereas in the case of animals previously inoculated with tuberculosis injections of TR modify the course of the disease and set up retrogressive changes. In man, immunising effects cannot be expected until doses of ½ to 1 milligramme are reached. No advantage can be expected in advanced cases, or in cases where there is evidence of mixed infection: but in suitable cases the expectoration diminishes and often disappears altogether, together with bacilli; dulness and crepitation also diminish in extent, and the only local symptom produced consists in a temporary increase of *râles*, whilst no disturbing constitutional symptoms have been observed.

Unfavourable reports have been published by Solles,[3] and

[1] *Lancet*, 1897, ii. 643.
[2] *Deutsche med. Wchnschr.*, 1897, xxiii. 209; abstract in *Practitioner*, 1897, lix. 399.
[3] *J. de Méd. de Bordeaux*, 1897, xxvii. 221; abstract in *Practitioner*, 1897, lix. 402.

several other observers comment on the difficulties in the method of dilution of the concentrated fluid; and they consider that at present the new tuberculin can only be used when it has been proved to be sterile, inasmuch as they found it to contain pneumococci, staphylococci, and streptococci. Another more serious charge against the TR is, that living tubercle bacilli may be obtained from it, and that the sediment obtained by centrifugalisation is capable of producing tuberculosis in guinea-pigs —as was shown to me by Dr. Baldwin at the Saranac Lake laboratory, where much useful investigation is being carried on by him and Dr. Trudeau.

Other observers have found no benefit from the use of this new tuberculin; so that from both points of view—(*a*) the utility of the preparation, (*b*) its safety—it seems necessary to wait for further evidence before admitting the remedy into general use for the treatment of tubercular diseases.

Hirschfelder's modified form of tuberculin is obtained by treating Koch's old tuberculin with peroxide of hydrogen.[1] This oxy-tuberculin, of which very large doses of 40 c.c. have been given, absolutely inhibits the growth of the bacillus in an alkaline culture medium.

Other methods of producing immunity against tuberculous infection are much sought after, but are difficult to attain. Dr. Paterson,[2] in his Weber-Parkes essay, describes a method of injection of the serum of immunised fowls for the purpose, and he believes that this condition of immunity can be obtained in man by the injection of 2 c.c. of his serum, increasing each administration by 1 c.c. until its influence on the body is shown by a distinct rise of temperature. He is unable to claim any curative effects from the use of the serum in patients suffering from tuberculous disease; but he holds, and that most emphatically, that when injected into susceptible bodies it confers on these an immunity against the invasion of the bacillus of mammalian tubercle; and he recommends that these injections should be given to persons who have a tuberculous tendency, and to those who have a history of tuberculous disease amongst their relatives.

Dr. J. H. Williams,[3] in a paper on eighteen years of personal observation of tuberculosis in Asheville, N.C., speaks strongly of the utility of the **antiphthisin** of Klebs. He states that the case books show that within the last two years there have been not less than thirty absolute and unequivocal recoveries under the administration of this remedy alone. Dr. S. Lane Anderson, after a report of a case in which eight bottles of Paquin's serum were used, states that he had never failed to see the benefit in seventy-two hours.[4] Dr. Allen, Kansas City, Mo., reports a

[1] *Med. News*, 1897, lxxi. 1; abstract in *Practitioner*, 1897, lix. 403.
[2] *Lancet*, 1897, ii. 1106.
[3] *J. Am. M. Ass.*, 1897, xxix. 363.
[4] *Ibid.*, 369.

recovery so complete that for two years not a single bacillus could be found.

Fraenkel[1] has treated thirty cases of tuberculosis with **ichthyol**, and after an experience of three months believes that the remedy acts much more energetically than creasote. He commences with two grammes per day, increasing to four at the end of a week. The cough, expectoration, and night-sweats in particular have been favourably influenced. It may be given in the form of the capsules "Raquin à l'ichthyol," or as keratin-coated tabloids. Further evidence of its utility is given by Dr. Williams, who, after using the remedy for eight years in daily doses of 20 to 100 minims, considers that it should rank not next to creasote but above it. In a large percentage of the cases he has observed a gradual involution and degeneration of the bacilli, with a reduction in number and their final disappearance.

R. SHINGLETON SMITH.

[1] *Therap. Wchnschr.*, April 11, 1897; abstract in *Practitioner*, 1897, lix. 407.

